# A review of popular vaping misconceptions: redefining ENDS safety and usage risks

**DOI:** 10.1080/08958378.2025.2571918

**Published:** 2025-10-15

**Authors:** Shaligram Sharma, Laura Crotty Alexander, Maureen Meister, Cassandra Ross, Joseph Hess, Kenneth Ray, Alexandra Noël, Jonathan Shannahan, Christa Wright

**Affiliations:** a Chemical Insights Research Institute, UL Research Institutes, Marietta, GA, USA;; b School of Medicine, University of California, San Diego, San Diego, CA, USA;; c Redan High School, Atlanta Public Schools, Atlanta, GA, USA;; d School of Health Sciences, Purdue University, West Lafayette, IN, USA;; e The Center for Black Health and Equity, Durham, NC, USA;; f Comparative Biomedical Sciences, Louisiana State University, Baton Rouge, LA, USA

**Keywords:** Vaping, safety, perceptions, misconceptions, ENDS, e-cigarettes

## Abstract

The widespread use of electronic nicotine delivery systems (ENDS) among youth and adults has become a significant public health concern. Approximately 19.6% of middle and high school students in the United States have reported using ENDS containing nicotine. Factors contributing to their popularity include social and recreational appeal, sensory satisfaction, ease of accessibility, and aggressive marketing strategies including influencer-driven promotions and youth-targeted campaigns through social media platforms. The variety of available flavors and modifiable features of ENDS devices further enhances their acceptance, often overshadowing their potential health risks. Despite their perceived advantages, misconceptions about ENDS persist, including beliefs that emissions are harmless, vaping is safer than smoking, and secondhand exposure is inconsequential. These misunderstandings contribute to the normalization of ENDS use, hindering public awareness of the associated health and environmental hazards. This manuscript addresses seven prevalent misconceptions about ENDS ranging from their safety during pregnancy to their environmental impact, highlighting the need for comprehensive education and community engagement to mitigate the risks of ENDS usage and promote informed decision-making. In the following section of the Special Issue Science Education and Research on Vaping and Interventions for Community Engagement (SERVICE), we will explore how these misconceptions not only encourage the societal acceptance and use of ENDS but also contribute to potential health risks.

## Introduction

Electronic nicotine delivery systems (ENDS) usage among youth and adults has become a serious health concern in developed nations. It is estimated that approximately 19.6% of middle and 4.7% of high school students in the United States have used ENDS devices containing nicotine (Wang et al. 2020). According to 2024 data from the Centers for Disease Control and Prevention (CDC), 1.63 million middle and high school students were active ENDS users within the 30 days preceding the survey (Park-Lee et al. 2024). The acceptance of ENDS devices over traditional cigarettes is often due to their perception as icebreakers for making friends, establishing communication, and being stylish and attractive. They are also popular among young people due to social, recreational, and sensory factors (Pokhrel et al. 2015; Cullen et al. 2018; Gentzke et al. 2020). Furthermore, the ease of accessibility, the influence of social media platforms like TikTok, Facebook, and Snapchat, and the variety of available flavors often overshadow the harmful effects of ENDS and their emissions (Sapru et al. 2020). The continually evolving landscape of emerging ENDS devices and products, with a wide range of modifiable device features (i.e. power settings, airflow, and coil resistance), has also contributed to their widespread acceptance (Ali et al. 2023; Tobacco Monitoring n.d). However, the long-term impact of ENDS on human health is not fully understood. This uncertainty is partly due to the limited historical perspective on long-term health effects of vaping and common misconceptions, such as the belief that these devices are free from nicotine, that vaping is safer, and that exhaled vaping emissions do not affect household members, pregnant women, and other bystanders. In the following sections, we attempt to address seven common misconceptions to raise public awareness about the real risks associated with ENDS usage.

Misconception 1: ENDS emissions are just water vapor.

Misconception 2: ENDS are safer than cigarettes.

Misconception 3: Vaping doesn’t hurt anyone around me.

Misconception 4: Vaping is safe during pregnancy.

Misconception 5: Vaping waste doesn’t pollute the environment.

Misconception 6: It is okay to dual use (ENDS, cigarettes, marijuana).

Misconception 7. Vaping is not addictive.

## Misconception 1: ENDS emissions are just water vapor

ENDS operate by heating e-liquid to high temperatures, causing the e-liquid to vaporize. While initially positioned as vapors by the vaping industry, ENDS emissions are not true vapors. Vapors are made up of individual molecules of a substance in gas form, like steam from boiling water, which fully mixes with air and leaves nothing behind. In contrast, ENDS emissions are aerosols, which is a suspension of tiny liquid or solid particles in air, often loaded with nicotine, metals, and other known and unknown chemicals (Ebrahimi Kalan et al. 2023; Omaiye Esther and Talbot 2025). This vapor is then inhaled by the user into their mouth and lungs, followed by its release into the environment as ENDS emissions (Ali et al. 2023). While e-liquids may appear to be simply viscous liquids mixed with or without flavoring agents like menthol, fruit extracts, and sweeteners to the untrained eye, they are far more complex. E-liquids contain an array of chemicals that may be disclosed on product labels if ingredient information is provided at all. For example, studies have shown that ENDS products can contain additional ingredients beyond what is disclosed on packaging, highlighting the uncertainty around their true chemical composition (Omaiye et al. 2019). A typical e-liquid contains nicotine, propylene glycol (PG), vegetable glycerin (VG), flavoring agents, and a variety of potentially harmful substances, including carcinogens (Tobacco Monitoring n.d; Feeney et al. 2022). PG and VG serve as carriers for nicotine and flavoring, with their ratio influencing both the flavor profile and the sensory experience of the user. Specifically, PG helps deliver flavors to the throat and provides a harsher taste, while VG is thicker, facilitating aerosol production and carrying the sweeter notes of the e-liquid (Feeney et al. 2022). The composition of the e-liquid alongside flavoring agents used to provide distinct flavors also influence the chemical profile of vaping emissions.

Apart from nicotine, both flavored and unflavored e-liquids significantly impact the chemical profile of vaping emissions. When subjected to thermal degradation, flavored e-liquids undergo chemical transformations, leading to the release of harmful and potentially carcinogenic agents such as formaldehyde, acetaldehyde, glyoxal, and the cardiovascular toxicant acrolein. These toxic aldehydes are known to cause oxidative stress, inflammation, and cytotoxicity, contributing to respiratory and cardiovascular diseases (Soneji et al. 2017; Ferkol et al. 2018; Hyde 2019). Furthermore, some flavor-specific toxic chemicals, particularly diacetyl and acetylpropionyl, detected in certain milk-, butter-, fruit-, candy, and cocktail-flavored e-liquids are commonly used to enhance sweetness and aroma. These diketones and other chemicals are well-documented respiratory toxicants linked to popcorn lung (bronchiolitis obliterans), a debilitating lung disease characterized by irreversible airway obstruction (Zhang et al. 2013; Eshraghian and Al-Delaimy 2021). Additionally, flavored e-liquid aerosols contain a higher number of chemical compounds compared to unflavored e-liquids, including volatile organic compounds (VOCs) such as benzene, toluene, ethylbenzene, and xylene (BTEX) (Zhang et al. 2013; Pinto et al. 2022; Hopstock et al. 2024). These compounds are associated with leukemia, neurotoxicity, and respiratory irritation, highlighting the increased toxic burden of flavored vaping emissions (Dibaji et al. 2018; Pinto et al. 2022; Hopstock et al. 2024). Furthermore, flavored e-liquids also generate higher concentrations of toxic metal nanoparticles due to interactions between flavoring agents and vaping device heating elements, such as nickel, chromium, and lead, which are known for their carcinogenic and neurotoxic effects (Sleiman et al. 2016; Kim et al. 2018; Vas et al. 2019). In contrast, unflavored e-liquids generate lower levels of these toxic emissions, reinforcing the direct role of flavoring agents in enhancing the chemical toxicity of vaping aerosols (Margham et al. 2021). Moreover, the American Cancer Society (ACS) has raised concerns about the risks of ‘dry puffing,’ a scenario in which formaldehyde from the heating element is released into ENDS aerosol when there is insufficient e-liquid in the pod or when the heating coil does not receive sufficient e-liquid (Omaiye Esther and Talbot 2025). Consequently, the composition and quantity of e-liquid are critical factors that affect both the performance of ENDS and the nature of the emissions produced. Specifically, ENDS emissions contain a complex array of known and unknown carcinogenic VOCs such as formaldehyde, heavy metals such as cadmium, chromium, and nickel, alongside ultrafine and fine particulates (Katz et al. 2019; Jeon et al. 2024; He et al. 2025). Given these findings, greater toxicological evaluation and regulatory oversight of flavored e-liquids are necessary to better understand and mitigate their long-term health impacts.

On the other hand, nicotine e-liquids may consist of nicotine salts or freebase nicotine, each with key differences in chemical structure, rate of absorption, and user experience (Leventhal et al. 2021). The use of nicotine salts can lead to higher nicotine delivery and can impact ENDS aerosol composition. Specifically, the inclusion of nicotine salts containing benzoic acid, can lead to free radical formation also known as reactive oxygen species (ROS) generation in ENDS aerosols that may offset critical biological processes causing oxidative stress (Barnett et al. 2024). Oxidative stress is an imbalance of ROS and innate antioxidants that are our natural defense to inhaled chemical hazards. Taken together, these points indicate that ENDS emissions are not simply water vapor but contain a range of harmful substances, found in the US Federal Drug Administration’s (FDA’s) International Agency for Research on Cancer (IARC) database. Therefore, it is essential to recognize that ENDS contain carcinogens and other toxic compounds that potentially contribute to cancer and other serious health conditions.

### Self-reflective questions for ENDS users or those considering using ENDS

- Do I know all the chemicals in my e-liquid?
- Is the nicotine content in my e-liquid accurate?
- How does heating my e-liquid affect its safety?
- What is the highest and lowest temperature my device can reach?
- Am I aware of the harmful byproducts from vaping?
- Do I know how e-liquids are regulated by FDA?
- Have I investigated recent research on e-liquids and their health effects?
- How transparent is the information about my e-liquid’s ingredients?
- What steps do I take to ensure my e-liquid is safe?

## Misconception 2: ENDS are safer than cigarettes

Since the introduction of ENDS in the US market, claims have been made that they are a safer alternative to cigarette smoking and can aid in smoking cessation (Feeney et al. 2022). However, safer does not mean safe, nor does it disregard the possibility that vaping could lead to riskier behavior, as evidenced by inconclusive scientific evidence to support these cessation claims (Ferkol et al. 2018; Hyde 2019). One systematic review and meta-analysis by Soneji et al. concluded that early exposure to vaping products may actually facilitate tobacco smoking initiation (Soneji et al. 2017). Since ENDS are relatively new products compared to combustible cigarettes, long-term epidemiological and toxicological data remain limited. In contrast to the lack of ENDS research, decades of scientific research unequivocally demonstrate that smoking is harmful, leading to the well-known conclusion that ‘Smoking Kills’ (Doll and Hill 1950). The misconception that vaping is safer than traditional cigarettes persist due to several factors, including the lack of substantial scientific information on the long-term and short-term health effects of vaping and the unknown composition of many e-liquids. Additionally, the ENDS market is unstable and rapidly evolving, with significant variability in device features and the range of e-liquids available, further complicating efforts to fully understand and regulate the health impacts of vaping products.

PG and VG are key components of e-liquids used in ENDS, alongside nicotine and food flavoring agents. While PG is effective in producing vapors, it can also lead to severe cough, eye irritation, lung injury, and may be linked to acquired asthma (Darabseh et al. 2020). Similarly, heating flavoring agents can produce carcinogenic chemicals that impair lung health, even though these agents are less harmful when ingested. For example, diacetyl, a common constituent of artificial butter flavoring, and safe for consumption, can result in bronchiolitis-like symptoms when heated and inhaled (Morgan et al. 2008; Zhang et al. 2013; Darabseh et al. 2020). Experiments on tissue explants and human lung cell lines exposed to ENDS emission extracts have shown increased cell death, DNA damage, and ROS production. The carcinogenic nature of these compounds is well documented, showing they cause DNA damage and inhibit repair mechanisms, conferring significant cancer risk (Shehata et al. 2023; de Oliveira et al. 2025). Although the toxicant profile of e-cigarettes remains insufficiently characterized, aldehyde exposure from vaping is substantial, and long-term cancer risk remains insufficiently defined (Sahu et al. 2023; Robertson et al. 2025; Toledo et al. 2025). Ongoing research and epidemiological surveillance are warranted. The authors would also like to note that other serious health conditions linked to vaping have already been published and are provided in the Special Issue manuscript, ‘Beyond the Puff: Health Consequences of Vaping’ (Meister et al. 2025).

In animal studies, rats exposed to PG at a concentration of 25 mg/kg/day for three days had observed respiratory arrest (Sun et al. 2024). Additionally, ENDS emissions generated from dessert-flavored e-liquids promote microbial adhesion to tooth enamel, demineralization, and biofilm formation, and has also been shown to contain metals such as cadmium, copper, iron, magnesium, and silicon (Sponziello et al. 2014; Hess et al. 2017; Kim et al. 2018). Exposure to nicotine and heavy metals from ENDS emissions over time can lead to impaired airway constriction, injury to epithelial linings, and an increased risk of wheezing and respiratory symptoms commonly reported by traditional smokers (Li et al. 2020). Studies have shown that ENDS emissions can cause DNA damage in the lungs, heart, and bladder, impair DNA repair mechanisms, and enhance mutation and transformation of tumorigenic cell types in cultured cells (Lee et al. 2018). Moreover, heating VG in the presence of other food additives or acids can result in pyrolysis and the production of chemicals such as acrolein, a nasal irritant that impairs lung function, causing symptoms such as wheezing, chest congestion, coughing, and eye irritation (Sun et al. 2024).

The collective evidence demonstrates that vaping is not safe and poses significant health risks especially for children, teens, and young adults (Health and Services 2016). The belief that vaping is a safer alternative to smoking is a misconception that needs to be addressed through clear scientific evidence and public health education. Recent research from Johns Hopkins has shown that emissions from disposable vaping devices can actually exceed those from traditional tobacco cigarettes, overturning earlier assumptions that ENDS produce lower emissions and are therefore safer (Martuzevicius et al. 2019; Aherrera et al. 2023; Mina et al. 2023). Furthermore, ENDS have not been conclusively proven to serve as effective smoking cessation tools (Lindson et al. 2025). It is essential to recognize and communicate the potential health hazards associated with vaping to dispel this dangerous misconception. Importantly, how vaping is framed—as ‘safer than smoking’ or compared instead to total abstinence—shapes public perception. Establishing the correct comparison point is therefore critical when developing effective health communication strategies.

### Self-reflective questions for ENDS users or those considering using ENDS

- Do I fully understand how my ENDS device works?
- Do I know the optimal temperature or wattage for operating my device?
- What type of e-liquid am I using, and how does it react to high heat?
- How aware am I of the flavoring chemicals in my e-liquid and their potential risks?
- Does my device track and monitor my usage?
- Am I informed about the health risks associated with vaping compared to smoking?
- Have I considered how vaping might impact my long-term health?
- What steps do I take to stay updated on the safety of my vaping products?

## Misconception 3: Vaping doesn’t hurt anyone around me

Vaping is frequently observed in outdoor spaces, social gatherings, among students in schools or colleges, and in enclosed areas (Katz et al. 2019). The exhaled vaping emissions linger in the air and can be unknowingly inhaled by bystanders by a process known as secondhand vaping (SHV), akin to environmental tobacco smoke or secondhand smoking (Protano and Vitali 2011). SHV emissions consisting of VOCs and particulates can accumulate on clothes, hair, dust, and furniture, leading to thirdhand vaping (THV) that can affect bystanders of all ages (Matt et al. 2023). It is crucial to dispel the misconception that vaping does not harm those nearby. Effective communication tools and messages from governments and funding organizations should clearly convey that vaping could potentially adversely affect everyone in close proximity and individuals who may encounter spaces or items exposed to hazardous ENDS emission components.

In a comprehensive study by Islam et al. the impact of SHV exposure among young adults was evaluated. The prevalence of SHV exposure was 11.7% at baseline, which increased to 15.6% over four years. This increase in SHV exposure was accompanied by an increase in wheezing, bronchitis-like symptoms, and shortness of breath (Islam et al. 2022). Similarly, Bayly et al. reported that increased SHV emissions are associated with a higher risk of asthma attacks in youth (Bayly et al. 2019). Recent, in-vitro, in-vivo, and population-based studies suggest that THV impairs lung function through inflammation and various cellular, molecular, and physiological alterations (Glynos et al. 2018). Study on animal models indicated that exposure to THV emissions significantly lowered serum IL-7 and increased bronchoalveolar lavage fluid IL-13, thereby impairing lung health (Commodore et al. 2023). Another study on mice exposed to vapors from PG/VG, PG/VG with nicotine, and flavored PG/VG with nicotine showed impaired lung cellular architecture. Specifically, exposure to PG/VG + nicotine + flavor increased MUC5ac, a protein that plays an important role in mucus production, oxidative biomarkers, and bronchoalveolar lavage fluid cellularity. PG/VG vapor exposure also impaired tissue elasticity, cellular static compliance, and airway resistance, with airway hyperreactivity being more pronounced when exposed to flavored PG/VG with nicotine (Glynos et al. 2018). This suggests that PG/VG, individually or combined with nicotine and flavors, detrimentally affects lung health.

Analysis of indoor air quality in homes of ENDS users and non-users revealed the presence of airborne nicotine particulates in vapers’ homes and were comparable to the emissions released in the homes of traditional smokers (Amalia et al. 2023; Cui et al. 2023). Non-users sharing a home with ENDS users had significant levels of cotinine, 3′-OH-cotinine, and 1,2-propanediol in saliva, as well as cobalt in urine, compared to non-users in control homes. This indicates that individuals can expose themselves to SHV and THV by living with vapers (Amalia et al. 2023). Vaping in homes or cars leads to the significant accumulation of nitrosamines and their derivatives on surfaces and materials, worsening indoor air quality and increasing the risk of SHV and THV exposure (Son et al. 2020; Soule et al. 2023). A study also highlighted that chemical ageing of exhaled emissions and its interaction with indoor atmospheric ozone may enhance inhalation toxicity due to the formation of radicals and alkyl hydroperoxides, which have greater oxidative potential and pose higher risks for SHV and THV (Woo et al. 2024).

Taken together, evidence indicates that ENDS aerosols contain significant amounts of chemicals and when mixed with environmental components they may produce harmful mixtures (Pinto et al. 2022; Salazar et al. 2025; Toledo et al. 2025). Particulate matter from the vapor may settle on objects that can act as reservoir. These reservoirs provide a constant source of exposure to the most vulnerable populations such as infants, elderly, and immunocompromised people (Schmidt 2020; Pinto et al. 2022). Infants are highly susceptible to these chemicals as their immature immune systems, heightened respiratory rates, incomplete skin barriers, and behaviors such as crawling and mouthing objects increase their exposure risk compared to adults (Pinto et al. 2022; Guraka et al. 2024).

These findings underscore the clear scientific consensus that vaping emissions can adversely affect everyone, with a critical emphasis on protecting vulnerable populations from the potential harmful effects of SHV and THV exposures.

### Self-reflective questions for ENDS users or those considering using ENDS

- Am I vaping inside, outside, or in a busy area?
- Who is near me when I vape?
- Am I transferring vape residues to my clothes and exposing others?
- Is my home safe for others if I vape inside?
- Am I aware of how vaping might affect those around me?
- Do I consider vulnerable people when I vape?
- Do I think about the vapor lingering in the environment?
- Are there rules about vaping where I am?
- Could my vaping habits affect others through my belongings?
- Do I know the risks of secondhand and thirdhand vaping exposures?

## Misconception 4: Vaping is safe during pregnancy

Epidemiological and gene-environmental interaction studies indicate that smoking during pregnancy may result in minor to permanent damage to the unborn child and potentially predispose epigenetic changes that increase susceptibility to certain diseases compared to their peers (Braun et al. 2020). Women with a history of smoking at reproductive ages have shown elevated levels of oxidative stress biomarkers, inflammation, and significant impacts on reproductive health. Increased levels of lead, nicotine metabolites, nitrosamines, and VOCs have been observed in these women (Perez et al. 2021). Although the health risks associated with smoking while pregnant are well known, vaping during pregnancy has emerged as a public health concern.

In a cross-sectional study, 11.9% of the 382 pregnant women participants reported using ENDS devices, while 30.3% used both ENDS and tobacco cigarettes during pregnancy. Participants reported having adequate information about the risks associated with ENDS; however, they perceived ENDS as less risky compared to traditional cigarettes and a tool for smoking cessation (Bhandari et al. 2018). Although the current evidence is limited and inconsistent, research has suggested that exclusive use of vaping products during pregnancy is associated with an increased risk of delivering a small-for-gestational-age infant compared to non-users (Wang et al. 2020; Shittu et al. 2022). Vaping while pregnant can cause a vast array of adverse effects not only for the mother but for the developing fetus as well. During early development, rapidly dividing cells are highly susceptible to changes in the composition of the mother’s blood. Thus, parental vaping is considered a major environmental risk factor that could affect a child’s growth (Braun et al. 2020). The failure of healthcare providers to evaluate ENDS usage during prenatal visits raises serious questions about the effectiveness of awareness efforts regarding the risks associated with ENDS (Bhandari et al. 2018). Nicotine is a known teratogen (Vaglenova et al. 2008) and the presence of comparable levels of cotinine, a metabolite of nicotine, in cord blood plasma among mothers and fetuses indicates that cotinine can be used as a biomarker for fetal exposure to maternal smoke. Cotinine crossing the placental barrier or being present in breast milk may result in spontaneous abortion or premature childbirth due to increased prostaglandin production, a hormone known to induce contractions during childbirth (Perez et al. 2021; Szukalska et al. 2021; Vilcassim et al. 2023). Heavy metals such as lead, cadmium, and mercury have been linked to an increased risk of abnormalities in the unborn child (Szukalska et al. 2021; Vilcassim et al. 2023). The impact of maternal vaping during pregnancy extends beyond fetal exposure to cotinine and metals, leading to behavioral and psychological effects in the child after birth. These children may exhibit increased aggressiveness, hyperactivity, and oppositional tendencies by 2–3 years of age (Abraham et al. 2017; Mescolo et al. 2021; Vilcassim et al. 2023; Ussher et al. 2024). In utero, physiological measurements are significantly impaired, such as femur length and head size (Abraham et al. 2017; Vilcassim et al. 2023). For non-vapers, exposure to ENDS emissions significantly transfers glycerol and metals like aluminum, chromium, nickel, copper, zinc, tin, and lead into the unborn child’s cord blood (Ballbè et al. 2023). *In vivo* studies by Cahill et al. on pregnant BALB/c mice exposed to flavored ENDS emissions showed altered lung alveologenesis, impaired Wnt signaling genes, and increased susceptibility to inflammation in the offspring. Female offspring exhibited a significant reduction in the methylation of the Il10ra gene promoter, while males developed strong pulmonary inflammation (Noël et al. 2020; Cahill et al. 2022; 2022). Beyond the lungs, neuroinflammation and behavioral modulation have also been reported in adult offspring of CD1 mice. ENDS aerosol-exposed mice showed significant negative impacts on brain health, with increased expression of inflammatory genes such as IL-6 (cerebellum), IL-4, and Interferon-gamma increased locomotor activity in plus maze experiments, and changes in stress-coping mechanisms (Church et al. 2020). Overall, there is a significant misconception that vaping is a safer alternative for pregnant and lactating women. However, preliminary scientific evidence affirms that the use of ENDS is not risk free and holds greater potential to harm maternal and fetal health. Therefore, it is necessary and critical to educate pregnant women about the harm associated with ENDS use.

### Self-reflective questions for ENDS users or those considering using ENDS

- Do I know what is in the ENDS emissions I am breathing?
- Do I know how my vaping habits might impact my reproductive health?
- Do I know how vaping emissions can harm my child’s development?
- Did I discuss my ENDS use and potential health impacts with my physician?
- Do I know the difference between vaping and smoking?
- Have I been asked about my use of nicotine or related products?

## Misconception 5: Vaping waste doesn’t pollute the environment

With the increasing popularity of single-use or rechargeable ENDS among all age groups, the amount of toxic and plastic waste generated has significantly risen (Truth Initiative 2022). Federal guidelines at manufacturing facilities prohibit open disposal of this waste and mandate a disposal management system. However, these standards are often disregarded, especially when users want to discard their ENDS. This contributes to the growing waste from disposable ENDS, designed to be discarded after a single use, thereby filling landfills. Each of these disposable devices contains nicotine-laden e-liquids, lithium batteries, heated metal coils, other heavy metals produced during vaping, and non-biodegradable plastic cases, all adding to environmental waste (Krause and Townsend 2015; Ngambo et al. 2023). According to the CDC, the number of vaping devices sold annually in the US, if lined up, would stretch over 7,000 miles—enough to span the continental US twice (Tobacco Monitoring n.d; CDC 2024). Lead and mercury from these devices can leach into soil and sand. Ocean conservancy and beach cleaning volunteers report a significant decline in cigarette butt waste but a significant increase in ENDS device waste, which endangers oceanic biodiversity due to non-biodegradable plastics and single-use components such as nicotine pods (Gutterman and Fund 2023). Poised with increased users and acceptability, ENDS could potentially become the number one waste item, persisting in nature without decomposing (Gutterman and Fund 2023; Donovan et al. 2025). For example, Krause et al. evaluated 23 disposable pods and found that lead leached at significantly high levels, measured by the California Waste Extraction Test (50 mg/L) and Toxicity Characteristic Leaching Procedure (40 mg/L). Of the pods evaluated, two products exceeded lead regulatory limits. Importantly, unused ENDS devices are categorized as hazardous waste (P075) due to nicotine content, highlighting concerns over the growing volume of discarded devices (Krause and Townsend 2015).

Beyond chemical leaching, the electronic components of ENDS pose substantial challenges for waste management. Recognizing this, the European Parliament classified battery-powered ENDS devices as waste electrical and electronic equipment in 2011–2012, emphasizing the need for comprehensive regulation addressing both the chemical and electronic waste components of ENDS. An online survey conducted by Truth Initiative on ENDS disposal methods revealed a widespread lack of knowledge among young people regarding proper disposal practices for pods and devices. Many respondents indicated that their initial instinct was to dispose of these items in regular trash bins, as recycling bins are not designated for ENDS. Manufacturers also do not provide clear guidance on proper disposal methods (Truth Initiative 2022; Gutterman and Fund 2023).

Federal, state, and local regulatory agencies primarily focus on the disposal of ENDS components, such as lithium-ion batteries and residual nicotine, rather than the entire device. Improper disposal in household trash or recycling streams can create fire hazards and environmental contamination (U.S. Food and Drug Administration 2024; United States Environmental Protection Agency 2025a). The US Environmental Protection Agency (EPA) and FDA recommend classifying ENDS components as hazardous waste, a position reinforced for schools and businesses under the Resource Conservation and Recovery Act (RCRA). Certain states, including California and Minnesota, mandate that ENDS waste be managed at designated collection sites with documented handling protocols (Minnesota Pollution Control Agency 2017; Recycling Partnership 2024; Recycling Today 2024; U.S. Food and Drug Administration 2024; United States Environmental Protection Agency 2025a, 2025b). In the absence of a standardized national recycling program, disposal practices vary by jurisdiction but consistently aim to isolate hazardous materials from standard waste streams to protect public health and the environment (EPA 2025; U.S. Food and Drug Administration n.d). Overall, studies by regulatory agencies and research centers indicate a significant lack of understanding among ENDS users regarding the environmental and health impacts of these devices. The improper classification of ENDS as mere vaping devices or electronic devices fails to address their actual composition and potential health and environmental risks.

### Self-reflective questions for ENDS users or those considering using ENDS

- Do I know how to dispose of my device properly?
- How is my e-cigarette/ENDS device powered, and do I know how to dispose of the battery?
- Can I identify the waste components of my device?
- Which parts of my device are recyclable plastics and which are single-use plastics?
- Am I familiar with federal guidelines for disposing of ENDS?
- How much e-liquid/nicotine was left in my device when I discarded it?
- Do I know where the nicotine from my discarded device is going? Is it possibly contaminating the environment?

## Misconception 6: It is okay to dual use (ENDS, cigarettes, marijuana)

The practice of combining ENDS with marijuana or traditional smoking has gained popularity under the mistaken belief that ‘dual use’ is harmless, or no worse than use of one inhalant alone. However, emerging scientific evidence challenges this notion, revealing significant health risks including cancer and exacerbated health conditions (Tang et al. 2021; Glantz et al. 2024; Ndeke et al. 2024). Recent studies have shown ENDS usage elicits 3.5 times greater risk of marijuana use particularly among adolescents and young adults (Chadi et al. 2019). A narrative literature review by Ndeke et al. highlighted that ENDS aerosols contain approximately 350 chemicals, including 14 VOCs and 30 newly formed chemicals (Ndeke et al. 2024). Controlled studies analyzing marijuana vapors have detected harmful substances such as acrolein, methacrolein, nine other degradation byproducts, and numerous ultrafine particulates. Notably, particulates from marijuana smoking can linger in the air for up to three hours (Tang et al. 2021). Furthermore, analysis of twelve cannabis ENDS cartridges from California’s surveillance program identified over 19 cannabinoids, 100 terpenes, natural extracts, and potentially toxic additives (Guo et al. 2021). These findings suggest that individuals who engage in dual use are exposed to a complex array of toxic substances, potentially leading to additive or synergistic health effects. In addition to chemical exposure, dual use is associated with various behavioral and psychological factors. Research indicates that adolescents who use both ENDS and traditional cigarettes or other tobacco products are 5.39 times more likely to use marijuana, compared to a 3.10 times higher likelihood among those who use ENDS alone (Lewis et al. 2022). Furthermore, exposure to ENDS advertisements significantly raises the probability of later using ENDS with marijuana, highlighting the role of marketing in driving dual-use behaviors (Kreitzberg et al. 2019). Among young adults aged 18–24 with prior marijuana use, 80.1% reported using a tobacco product or device for marijuana consumption, with ENDS accounting for 24.5% of these cases (Rauh and Margolis 2016), suggesting substantial health risk associated with dual use.

With the increasing legalization of cannabis, dual use of ENDS and marijuana has become more prevalent, raising important public health concerns. A clinical case report by Chua et al. identified a possible link between concurrent cannabis and ENDS use and the development of pulmonary alveolar proteinosis (PAP), suggesting a potential risk for autoimmune PAP disease associated with vaping (Soule et al. 2023). Additionally, a systematic review by Pisinger and Rasmussen, supported by a six-year follow-up study, found that dual use is as harmful as traditional smoking, with dual users facing significantly higher health risks than conventional smokers (Guraka et al. 2024). These observations highlight the considerable risks of dual use, though further research is needed to clarify interactions between marijuana and ENDS particulates.

Beyond health concerns, several factors influence dual use patterns, including motivation, recreational habits, cessation strategies, and cognitive factors. Initially marketed as a smoking cessation aid, ENDS have not proven effective in long-term smoking cessation efforts (Chirumamilla et al. 2012; Meghea 2020; Kasza et al. 2021; Coleman et al. 2022). Instead, they have provided smokers with an additional option that can be used in environments where traditional smoking is prohibited or socially discouraged, owing to legal permissibility and social acceptance (Landry et al. 2019; Agaku et al. 2022; Temourian et al. 2022). However, research indicates that ENDS are effective primarily in short-term cessation programs, with no clear evidence linking dual use to sustained tobacco cessation (Sweet et al. 2019; Romberg et al. 2021).

Further research is essential to explore how chemicals and particulates stemming from marijuana use and vaping may interact synergistically to increase health risks. Understanding these interactions and the broader implications of dual product use is crucial for developing effective public health strategies.

### Self-reflective questions for ENDS users or those considering using ENDS

- Am I aware that dual use exposes you to harmful chemicals?
- Do I know how long harmful chemicals from vaping can linger in the air?
- Does evidence show ENDS guarantee long-term success in quitting smoking?
- Am I aware that dual use doesn’t help to quit smoking?
- Do I know of counseling or nicotine replacement therapy options?

## Misconception 7: Vaping is not addictive

The use of ENDS devices is associated with serious health concerns, particularly regarding their potential for addiction and psychological distress (Lohner et al. 2023). In a study by Jankowski et al. the Fagerström Test for Nicotine Dependence (FTND) measured nicotine dependency among smokers, ENDS users, and dual users. The findings revealed that e-cigarette users exhibited a nicotine dependency index twice as high as traditional smokers, with dual users showing an even greater dependency (Jankowski et al. 2019). Key signs of addiction include vaping early in the morning, a strong urge to vape, and daily use. Approximately 17% of ENDS users reported feeling very addicted, 35% considered ENDS equally addictive as tobacco cigarettes, and about 6% felt ENDS were more addictive than tobacco cigarettes (Lohner et al. 2023). These results highlight the urgent need for addressing the addictive nature of ENDS and dual use, along with whether cessation strategies should include ENDS (Jankowski et al. 2019).

Nicotine dependency can worsen among users motivated to use ENDS devices as a cessation strategy, leading to increased usage (Temourian et al. 2022). Additionally, transitioning from traditional smoking to ENDS often results in higher nicotine and e-liquid consumption, further propagating addiction instead of promoting cessation (Zavala-Arciniega et al. 2021). A study by Wang and colleagues found that single or dual users of ENDS are more likely to experience moderate to severe psychological distress compared to nonsmokers. The severity of distress varies with smoking habits; for instance, dual users who smoke daily are more likely to experience severe distress (Wang et al. 2023). Overall, these findings suggest that whether someone uses ENDS individually or dually is associated with the severity of psychological distress, and the frequency of smoking determines the levels of severity (Wang et al. 2023).

Nicotine formulations in ENDS have evolved substantially, with nicotine salts now representing the predominant form in most e-liquids. These salts are produced by combining nicotine with organic acids (e.g. benzoic acid) to lower pH, creating a smoother aerosol that reduces throat irritation and facilitates-inhalation of higher nicotine doses (Etter and Eissenberg 2015; Jankowski et al. 2019; Glantz et al. 2022). Such properties allow users, particularly adolescents and nicotine-naïve- individuals, to tolerate high nicotine concentrations sometimes exceeding 60 mg/mL thereby increasing systemic exposure and risk of dependence (Cho et al. 2024; Harris 2024). In combination with modern ENDS device technology designed to optimize aerosol delivery, nicotine salt formulations contribute to prolonged use, greater nicotine intake, and potentially stronger dependence patterns (Cho et al. 2024; Harris 2024). Although some former smokers report perceiving ENDS dependence as ‘weaker’ than cigarette dependence, evidence suggests that salt-based e-liquids may enhance addiction vulnerability in young adults and inexperienced users (Etter and Eissenberg 2015; Kempf et al. 2017; Cousijn et al. 2018; Glantz et al. 2022; Cho et al. 2024).

In addition to nicotine salts, newer formulations such as synthetic nicotine and analogs like 6-methylnicotine are appearing on the market. Synthetic nicotine, available as racemic mixtures or as enantiomerically pure compounds, is often marketed as ‘tobacco free’- and has been used to circumvent existing tobacco product regulations, raising novel policy and toxicological concerns (Berman et al. 2023; Jordt 2023; Jordt et al. 2025). Nicotine analogs such as 6-methylnicotine demonstrate even greater potency at nicotinic acetylcholine receptors in experimental models, suggesting the potential for increased addiction liability compared to nicotine itself (Erythropel et al. 2024; Jabba and Jordt 2024; Pankow et al. 2025). These developments underscore the importance of evaluating both established formulations such as nicotine salts and emerging synthetic or analog products to fully understand the evolving risks of ENDS use (Berman et al. 2023; Cheetham et al. 2023). Overall, while traditional cigarettes have well-documented health risks, the study of ENDS addiction and its health impacts is limited. It is essential to evaluate the full spectrum of health impacts associated with these newer devices (Jankowski et al. 2019).

### Self-reflective questions for ENDS users or those considering using ENDS

- Am I aware that ENDS can be just as addictive as traditional cigarettes?
- Do I crave ENDS first thing in the morning?
- Do I believe ENDS are safer than traditional cigarettes if they still cause addiction?
- Am I aware of the mental health impacts of daily ENDS use?
- Am I aware new nicotine delivery methods in ENDS can heighten addiction?

In conclusion, while some studies indicate ENDS may be less addictive than traditional cigarettes for some users, they are not without significant addictive potential. The presence of nicotine and observed patterns of use and dependence highlight the need to treat ENDS as addictive substances, considering their impact on public health, especially among young people. The addiction potential of ENDS is influenced by factors such as nicotine concentration, device type, and duration of use. Additionally, the formulation of e-liquids, including nicotine concentration, pH level, and flavors, plays a crucial role. The trend toward more efficient nicotine delivery systems and appealing flavors raises concerns about increased addiction risk, particularly among younger users.

## Conclusions

ENDS emissions are far from harmless water vapor and instead contain a complex mixture of VOCs, heavy metals, and carcinogens that pose substantial health risks to both users and bystanders through SHV and THV exposure.

E-liquids, despite their seemingly simple composition of nicotine, flavoring agents, and PG/VG, undergo chemical transformations when heated, generating toxic aerosols that linger in the air, accumulate on surfaces, and contribute to long-term environmental contamination, disproportionately affecting vulnerable populations such as infants, the elderly, and immunocompromised individuals. Scientific research has conclusively refuted the assumption that vaping during pregnancy is safe, linking prenatal ENDS exposure to fetal growth restrictions, neurodevelopmental deficits, and immune dysfunction, while also highlighting the alarming lack of awareness among healthcare providers regarding these risks.

Engraving the concern is the belief that combining ENDS with marijuana or traditional cigarettes or other forms of noncombustible products does not amplify harm. In contrast, studies demonstrate that dual users face equal or greater health risks than conventional smokers, which can exacerbate respiratory and immune dysfunction. The notion that ENDS serve as an effective smoking cessation tool is controversial (Conde et al. 2024), as evidence indicates that ENDS users often exhibit higher nicotine dependence than traditional smokers (Bandi et al. 2023), with protonated nicotine formulations facilitating rapid absorption and reinforcing addiction, thereby undermining their potential as a harm-reduction strategy (Gholap et al. 2020; Cho et al. 2024). Moreover, the assumption that vaping is environmentally safe fails to account for the staggering rise in toxic and plastic waste generated by disposable and rechargeable ENDS, which contain lithium-ion batteries, metal coils, and non-biodegradable plastics that leach hazardous substances such as lead and mercury into soil and water, exacerbating ecological damage. The widespread lack of public awareness regarding proper disposal practices further intensifies this environmental crisis, as the majority of users continue to discard ENDS devices irresponsibly, potentially accelerating pollution and biodiversity loss.

Ultimately, the persistent misconceptions surrounding ENDS contribute to the widespread under-estimation of their multifaceted risks, underscoring the urgent need for stringent regulatory policies, targeted public education initiatives, and continued scientific investigation to fully elucidate their long-term health and environmental consequences.

## Data Availability

Data sharing is not applicable to this article as no new data were created or analyzed in this study.

## References

[R1] Abraham M 2017. A systematic review of maternal smoking during pregnancy and fetal measurements with meta-analysis. PLoS One. 12(2):e0170946. 10.1371/journal.pone.0170946 28231292 PMC5322900

[R2] Agaku I, Egbe CO, Ayo-Yusuf O. 2022. Associations between electronic cigarette use and quitting behaviours among South African adult smokers. Tob Control. 31(3):464–472. 10.1136/tobaccocontrol-2020-056102 33452210

[R3] Aherrera A 2023. Metal concentrations in e-cigarette aerosol samples: a comparison by device type and flavor. Environ Health Perspect. 131(12):127004. 10.1289/EHP11921 38048100 PMC10695266

[R4] Ali FR, S A, Crane E, Seaman E, Tynan MA, Marynak K. 2023. E-cigarette unit sales by product and flavor type, and top-selling brands, United States, 2020–2022. MMWR Morb Mortal Wkly Rep. 72(25):672–677. 37347717 10.15585/mmwr.mm7225a1 PMC10328473

[R5] Amalia B 2023. Exposure to secondhand aerosol from electronic cigarettes at homes: a real-life study in four European countries. Sci Total Environ. 854:158668. 10.1016/j.scitotenv.2022.158668 36099951

[R6] Ballbè M 2023. Passive exposure to electronic cigarette aerosol in pregnancy: a case study of a family. Environ Res. 216(Pt 1):114490. 10.1016/j.envres.2022.114490 36220444

[R7] Bandi P 2023. Changes in e-cigarette use among U.S. adults, 2019–2021. Am J Prev Med. 65(2):322–326. 10.1016/j.amepre.2023.02.026 37479423

[R8] Barnett L 2024. 3D printer emissions elicit filament-specific and dose-dependent metabolic and genotoxic effects in human airway epithelial cells. Front Public Health. 12:1408842. 10.3389/fpubh.2024.1408842 39071151 PMC11273288

[R9] Bayly JE 2019. Secondhand exposure to aerosols from electronic nicotine delivery systems and asthma exacerbations among youth with asthma. Chest. 155(1):88–93. 10.1016/j.chest.2018.10.005 30359612 PMC6688978

[R10] Berman ML, Zettler PJ, Jordt SE. 2023. Synthetic nicotine: science, global legal landscape, and regulatory considerations. World Health Organ Tech Rep Ser. 1047:35–60. 37745838 PMC10516533

[R11] Bhandari NR 2018. Use and risk perception of electronic nicotine delivery systems and tobacco in pregnancy. Womens Health Issues. 28(3):251–257. 10.1016/j.whi.2018.02.005 29588116

[R12] Braun M 2020. Influence of second-hand smoke and prenatal tobacco smoke exposure on biomarkers, genetics and physiological processes in children—an overview in research insights of the last few years. Int J Environ Res Public Health. 17(9):3212. 10.3390/ijerph17093212 32380770 PMC7246681

[R13] Cahill KM 2022. In utero exposure to electronic-cigarette aerosols decreases lung fibrillar collagen content, increases Newtonian resistance and induces sex-specific molecular signatures in neonatal mice. Toxicol Res. 38(2):205–224. 10.1007/s43188-021-00103-3 35415078 PMC8960495

[R14] Cahill KM 2022. In utero exposures to mint-flavored JUUL aerosol impair lung development and aggravate house dust mite-induced asthma in adult offspring mice. Toxicology. 477:153272. 10.1016/j.tox.2022.153272 35878681 PMC9706914

[R15] CDC. 2024. About E-cigarettes (vapes). https://www.cdc.gov/tobacco/e-cigarettes/about.html

[R16] Chadi N 2019. Association between electronic cigarette use and marijuana use among adolescents and young adults: a systematic review and meta-analysis. JAMA Pediatr. 173(10):e192574. 10.1001/jama-pediatrics.2019.2574 31403684 PMC6692686

[R17] Cheetham A 2023. Chemical, pharmacological, and toxicological assessment of 6-methylnicotine. Brain. 3:7.

[R18] Chirumamilla AP 2012. High platelet reactivity on clopidogrel therapy correlates with increased coronary atherosclerosis and calcification: a volumetric intravascular ultrasound study. JACC Cardiovasc Imaging. 5(5): 540–549. 10.1016/j.jcmg.2011.12.019 22595163 PMC3753810

[R19] Cho YJ 2024. E-cigarette nicotine delivery among young adults by nicotine form, concentration, and flavor: a crossover randomized clinical trial. JAMA Netw Open. 7(8):e2426702. 10.1001/jamanetworkopen.2024.26702 39120901 PMC11316233

[R20] Church JS 2020. Neuroinflammatory and behavioral outcomes measured in adult offspring of mice exposed prenatally to e-cigarette aerosols. Environ Health Perspect. 128(4):47006. 10.1289/EHP6067 32293200 PMC7228099

[R21] Coleman SRM 2022. Dual use of combustible cigarettes and e-cigarettes: a narrative review of current evidence. Curr Addict Rep. 9(4):353–362. 10.1007/s40429-022-00448-1 36467719 PMC9718538

[R22] Commodore S 2023. Thirdhand vaping exposures are associated with pulmonary and systemic inflammation in a mouse model. J Environ Expo Assess. 2(4):22. 10.20517/jeea.2023.27 38741701 PMC11090496

[R23] Conde M 2024. Electronic cigarettes and subsequent use of cigarettes in young people: an evidence and gap map. Addiction. 119(10):1698–1708. 10.1111/add.16583 38937796 PMC12146478

[R24] Cousijn J, Luijten M, Feldstein Ewing SW. 2018. Adolescent resilience to addiction: a social plasticity hypothesis. Lancet Child Adolesc Health. 2(1):69–78. 10.1016/S2352-4642(17)30148-7 30169197 PMC6373770

[R25] Cui T 2023. PM1 exposure and spatial transmission of nicotine from the simulated second-hand vapor of pod-based electronic cigarettes. Sci Total Environ. 897: 165355. 10.1016/j.scitotenv.2023.165355 37419341

[R26] Cullen KA 2018. Use of electronic cigarettes and any tobacco product among middle and high school students—United States, 2011–2018. MMWR Morb Mortal Wkly Rep. 67(45):1276–1277. 10.15585/mmwr.mm6745a5 30439875 PMC6290807

[R27] Darabseh MZ 2020. Is vaping better than smoking for cardiorespiratory and muscle function? Multidiscip Respir Med. 15(1):674. 10.4081/mrm.2020.674 32670575 PMC7348661

[R28] de Oliveira GG 2025. Evidence on vaping e-cigarettes as a risk factor for cancer: a systematic review. J Cancer Policy. 45:100615. 10.1016/j.jcpo.2025.100615 40691942

[R29] Dibaji SAR 2018. Accuracy of commercial electronic nicotine delivery systems (ENDS) temperature control technology. PLoS One. 13(11):e0206937. 10.1371/journal.pone.0206937 30395592 PMC6218080

[R30] Doll R, Hill AB. 1950. Smoking and carcinoma of the lung; preliminary report. Br Med J. 2(4682):739–748. 10.1136/bmj.2.4682.739 14772469 PMC2038856

[R31] Donovan EM 2025. Not-so-disposable e-cigarettes: methods young people use to discard single-use e-cigarettes. Addiction. 120(3):452–457. 10.1111/add.16538 38828645

[R32] Ebrahimi Kalan M 2023. Terms tobacco users employ to describe e-cigarette aerosol. Tob Control. 33(1):15–20. 10.1136/tobaccocontrol-2021-057233 35728932 PMC9768092

[R33] EPA. 2025. [cited 2025 Aug 16]. Available from: https://www.epa.gov/hw/how-safely-dispose-e-cigarettes-information-individuals

[R34] Erythropel HC 2024. Variability in constituents of E-cigarette products containing nicotine analogues. JAMA. 332(9):753–755. 10.1001/jama.2024.12408. 39110443 PMC11307159

[R35] Eshraghian EA, Al-Delaimy WK. 2021. A review of constituents identified in e-cigarette liquids and aerosols. Tob Prev Cessat. 7(February):10–15. 10.18332/tpc/131111 33585727 PMC7873740

[R36] Etter JF, Eissenberg T. 2015. Dependence levels in users of electronic cigarettes, nicotine gums and tobacco cigarettes. Drug Alcohol Depend. 147:68–75. 10.1016/j.drugalcdep.2014.12.007 25561385 PMC4920051

[R37] Feeney S, Rossetti V, Terrien J. 2022. E-cigarettes-a review of the evidence-harm versus harm reduction. Tob Use Insights. 15:1179173–x221087524. 10.1177/1179173X221087524 PMC8968985 35370428

[R38] Ferkol TW 2018. Electronic cigarette use in youths: a position statement of the Forum of International Respiratory Societies. Eur Respir J. 51(5):1800278. 10.1183/13993003.00278-2018 29848575

[R39] Gentzke AS 2020. Tobacco product use among middle and high school students—United States, 2020. MMWR Morb Mortal Wkly Rep. 69(50):1881–1888. 10.15585/mmwr.mm6950a1 33332300 PMC7745956

[R40] Gholap VV 2020. Nicotine forms: why and how do they matter in nicotine delivery from electronic cigarettes? Expert Opin Drug Deliv. 17(12):1727–1736. 10.1080/17425247.2020.1814736 32842785 PMC9361466

[R41] Glantz S, Jeffers A, Winickoff JP. 2022. Nicotine addiction and intensity of e-cigarette use by adolescents in the US, 2014 to 2021. JAMA Netw Open. 5(11):e2240671. 10.1001/jamanetworkopen.2022.40671 36342713 PMC9641541

[R42] Glantz SA, Nguyen N, Silva A. 2024. Population-based disease odds for e-cigarettes and dual use versus cigarettes. NEJM Evid. 3(3):EVIDoa2300229. 10.1056/EVIDoa2300229 PMC11562742 38411454

[R43] Glynos C 2018. Comparison of the effects of e-cigarette vapor with cigarette smoke on lung function and inflammation in mice. Am J Physiol Lung Cell Mol Physiol. 315(5):L662–L672. 10.1152/ajplung.00389.2017 30091379

[R44] Guo W 2021. Major constituents of cannabis vape oil liquid, vapor and aerosol in California vape oil cartridge samples. Front Chem. 9:694905. 10.3389/fchem.2021.694905 34368078 PMC8333608

[R45] Guraka A 2024. A comprehensive toxicological analysis of panel of unregulated e-cigarettes to human health. Toxicology. 509:153964. 10.1016/j.tox.2024.153964 39362579

[R46] Gutterman LR, Fund UPE. 2023. Vape waste. https://publicinterestnetwork.org/wp-content/uploads/2023/07/Vape-Waste-Full-Report-PIRG.pdf

[R47] Harris T 2024. Physical and chemical characterization of aerosols produced from experimentally designed nicotine salt-based e-liquids. Chem Res Toxicol. 37(8):1315–1328. 10.1021/acs.chemrestox.4c00073 39078024 PMC11337207

[R48] He X 2025. Multi-omics assessment of puff volume-mediated salivary biomarkers of metal exposure and oxidative injury associated with electronic nicotine delivery systems. Environ Health Perspect. 133(1):17005. 10.1289/EHP14321 39819025 PMC11737583

[R49] Health U, Services H. 2016. E-cigarette use among youth and young adults: a report of the surgeon general. Atlanta (GA): Centers for Disease Control and Prevention (US). 30869850

[R50] Hess CA 2017. E-cigarettes as a source of toxic and potentially carcinogenic metals. Environ Res. 152:221–225. 10.1016/j.envres.2016.09.026 27810679 PMC5135636

[R51] Hopstock KS 2024. Chemical analysis of exhaled vape emissions: unraveling the complexities of humectant fragmentation in a human trial study. Chem Res Toxicol. 37(6):1000–1010. 10.1021/acs.chemrestox.4c00088 38769630 PMC11187636

[R52] Hyde C 2019. Usage of electronic cigarettes among youth in the United States. Ballard Brief. 2019(3):6.

[R53] Islam T 2022. Secondhand nicotine vaping at home and respiratory symptoms in young adults. Thorax. 77(7):663–668. 10.1136/thoraxjnl-2021-217041 35013000 PMC9203939

[R54] Jabba SV, Jordt SE. 2024. Marketing of nicotinamide as nicotine replacement in electronic cigarettes and smokeless tobacco. Tob Prev Cessat. 10(August):1–5. 10.18332/tpc/187767 PMC11295357 39132445

[R55] Jankowski M 2019. E-cigarettes are more addictive than traditional cigarettes—a study in highly educated young people. Int J Environ Res Public Health. 16(13): 2279. 10.3390/ijerph16132279 31252671 PMC6651627

[R56] Jeon J 2024. The role of puff volume in vaping emissions, inhalation risks, and metabolic perturbations: a pilot study. Sci Rep. 14(1):18949. 10.1038/s41598-024-69985-1 39147784 PMC11327287

[R57] Jordt S 2025. 6-Methyl nicotine in electronic cigarettes: chemical analysis and toxicological properties. Am J Respir Crit Care Med. 211(Abstracts):A7552–A7552. 10.1164/ajrccm.2025.211.Abstracts.A7552

[R58] Jordt S-E. 2023. Synthetic nicotine has arrived. BMJ Publishing Group Ltd. 10.1136/tobaccocontrol-2021-056626 PMC8898991 34493630

[R59] Kasza KA 2021. Association of e-cigarette use with discontinuation of cigarette smoking among adult smokers who were initially never planning to quit. JAMA Netw Open. 4(12):e2140880–e2140880. 10.1001/jamanetworkopen.2021.40880 34962556 PMC8715340

[R60] Katz SJ 2019. Beliefs about e-cigarettes: a focus group study with college students. Am J Health Behav. 43(1): 76–87. 10.5993/AJHB.43.1.7 30522568 PMC7015258

[R61] Kempf C 2017. What’s new in addiction prevention in young people: a literature review of the last years of research. Front Psychol. 8:1131. 10.3389/fpsyg.2017.01131 28729846 PMC5498551

[R62] Kim SA 2018. Cariogenic potential of sweet flavors in electronic-cigarette liquids. PLoS One. 13(9):e0203717. 10.1371/journal.pone.0203717 30192874 PMC6128655

[R63] Krause MJ, Townsend TG. 2015. Hazardous waste status of discarded electronic cigarettes. Waste Manag. 39:57–62. 10.1016/j.wasman.2015.02.005 25746178

[R64] Kreitzberg DS 2019. Exposure to ENDS advertising and use of marijuana in ENDS among college students. Addict Behav. 93:9–13. 10.1016/j.addbeh.2019.01.012 30677567 PMC6488373

[R65] Landry RL 2019. The role of flavors in vaping initiation and satisfaction among U.S. adults. Addict Behav. 99:106077. 10.1016/j.addbeh.2019.106077 31437770 PMC6903386

[R66] Lee H-W 2018. E-cigarette smoke damages DNA and reduces repair activity in mouse lung, heart, and bladder as well as in human lung and bladder cells. Proc Natl Acad Sci U S A. 115(7):E1560–E1569. 10.1073/pnas.1718185115 29378943 PMC5816191

[R67] Leventhal AM 2021. Effect of exposure to e-cigarettes with salt vs free-base nicotine on the appeal and sensory experience of vaping: a randomized clinical trial. JAMA Netw Open. 4(1):e2032757. 10.1001/jama-networkopen.2020.32757 33433597 PMC7804919

[R68] Lewis NM 2022. Characteristics of adults who use both marijuana and e-cigarette, or vaping, products: a cross-sectional study, Utah, 2018. Public Health Rep. 137(4):695–701. 10.1177/00333549211018679 34039118 PMC9257507

[R69] Li D 2020. Association of smoking and electronic cigarette use with wheezing and related respiratory symptoms in adults: cross-sectional results from the Population Assessment of Tobacco and Health (PATH) study, wave 2. Tob Control. 29(2):140–147. 10.1136/tobaccocontrol-2018-054694 30760629 PMC6692241

[R70] Lindson N 2025. Electronic cigarettes for smoking cessation. Cochrane Database Syst Rev. 1(1):Cd010216. 10.1002/14651858.CD010216.pub9 PMC11776059 39878158

[R71] Lohner V 2023. Understanding perceived addiction to and addictiveness of electronic cigarettes among electronic cigarette users: a cross-sectional analysis of the International Tobacco Control Smoking and Vaping (ITC 4CV) England Survey. Addiction. 118(7):1359–1369. 10.1111/add.16162 36772958 PMC10357132

[R72] Margham J 2021. The chemical complexity of e-cigarette aerosols compared with the smoke from a tobacco burning cigarette. Front Chem. 9:743060. 10.3389/fchem.2021.743060 34660535 PMC8514950

[R73] Martuzevicius D 2019. Characterization of the spatial and temporal dispersion differences between exhaled e-cigarette mist and cigarette smoke. Nicotine Tob Res. 21(10):1371–1377. 10.1093/ntr/nty121 29924352 PMC6751519

[R74] Matt GE 2023. Policy-relevant differences between secondhand and thirdhand smoke: strengthening protections from involuntary exposure to tobacco smoke pollutants. BMJ Publishing Group Ltd. 10.1136/tc-2023-057971 PMC11503167 37263783

[R75] Meghea CI. 2020. Electronic nicotine delivery systems use and cigarette smoking frequency and intensity among young adult smokers. JAMA Netw Open. 3(11): e2016121–e2016121. 10.1001/jamanetwor-kopen.2020.16121 33231632

[R76] Meister M 2025. Beyond the puff: health consequences of vaping. Inhal Toxicol. 1–14. 10.1080/08958378.2025.2500646 40367291

[R77] Mescolo F, Ferrante G, La Grutta S. 2021. Effects of e-cigarette exposure on prenatal life and childhood respiratory health: a review of current evidence. Front Pediatr. 9:711573. 10.3389/fped.2021.711573 34513764 PMC8430837

[R78] Mina WT 2023. Arsenic and arsenic species in MOD, POD, and disposable POD electronic cigarette aerosols: a pilot study. J Environ Expo Assess. 2(2):9. 39252696 10.20517/jeea.2023.03 PMC11382129

[R79] Minnesota Pollution Control Agency. 2017. Metropolitan solid waste management policy plan, 2022–2042. https://www.pca.state.mn.us/sites/default/files/w-sw7-22.pdf

[R80] Morgan DL 2008. Respiratory toxicity of diacetyl in C57BL/6 mice. Toxicol Sci. 103(1):169–180. 10.1093/toxsci/kfn016 18227102 PMC2669658

[R81] Ndeke JM, Klaunig JE, Commodore S. 2024. Nicotine or marijuana vaping exposure during pregnancy and altered immune responses in offspring. J Environ Expo Assess. 3(1):. 10.20517/jeea.2024.03 PMC11152453 38840831

[R82] Ngambo G 2023. A scoping review on e-cigarette environmental impacts. Tob Prev Cessat. 9(October):30–38. 10.18332/tpc/172079 37789930 PMC10542855

[R83] Noël A 2020. In utero exposures to electronic-cigarette aerosols impair the Wnt signaling during mouse lung development. Am J Physiol Lung Cell Mol Physiol. 318(4):L705–L722. 10.1152/ajplung.00408.2019 32083945

[R84] Omaiye EE 2019. High-nicotine electronic cigarette products: toxicity of JUUL fluids and aerosols correlates strongly with nicotine and some flavor chemical concentrations. Chem Res Toxicol. 32(6):1058–1069. 10.1021/acs.chemrestox.8b00381 30896936 PMC6579667

[R85] Omaiye Esther E, Talbot P. 2025. Quantification of 16 metals in fluids and aerosols from ultrasonic pod-style cigarettes and comparison to electronic cigarettes. Environ Health Perspect. 133(5):57020. 10.1289/EHP15648 40207990 PMC12118356

[R86] Pankow JF 2025. Levels of the nicotine analog 6-methyl nicotine as a naturally formed tobacco alkaloid in tobacco and tobacco products. Sci Rep. 15(1):17945. 10.1038/s41598-025-01392-6 40410306 PMC12102161

[R87] Park-Lee E 2024. Notes from the field: e-cigarette and nicotine pouch use among middle and high school students - United States, 2024. MMWR Morb Mortal Wkly Rep. 73(35):774–778. 10.15585/mmwr.mm7335a3 39236021 PMC11376506

[R88] Perez MF 2021. Biomarkers of toxicant exposure and inflammation among women of reproductive age who use electronic or conventional cigarettes. J Womens Health (Larchmt). 30(4):539–550. 10.1089/jwh.2019.8075 33534627 PMC8064962

[R89] Pinto MI 2022. Chemical characterisation of the vapour emitted by an e-cigarette using a ceramic wick-based technology. Sci Rep. 12(1):16497. 10.1038/s41598-022-19761-w 36192548 PMC9529894

[R90] Pokhrel P 2015. Young adult e-cigarette users’ reasons for liking and not liking e-cigarettes: a qualitative study. Psychol Health. 30(12):1450–1469. 10.1080/08870446.2015.1061129 26074148 PMC4657726

[R91] Protano C, Vitali M. 2011. The new danger of thirdhand smoke: why passive smoking does not stop at second-hand smoke. Environ Health Perspect. 119(10):A422. 10.1289/ehp.1103956 PMC3230455 21968336

[R92] Rauh VA, Margolis AE. 2016. Research review: environmental exposures, neurodevelopment, and child mental health - new paradigms for the study of brain and behavioral effects. J Child Psychol Psychiatry. 57(7):775–793. 10.1111/jcpp.12537 26987761 PMC4914412

[R93] Recycling Partnership. 2024. Report shows only 21% of U.S. residential recyclables are captured; points to policy and investment as immediate solutions. https://recyclingpartner-ship.org/report-shows-only-21-of-u-s-residential-recyclables-are-captured-points-to-policy-and-investment-as-immediate-solutions/

[R94] Recycling Today. 2024. Protecting against LIB fires in recycling facilities. https://www.recyclingtoday.com/news/protecting-against-lithium-ion-battery-fires-in-recycling-facilities/

[R95] Robertson NE 2025. E-liquid and aerosol characterization of popular disposable e-cigarettes. ACS Omega. 10(27): 29615–29627. 10.1021/acsomega.5c03167 40687050 PMC12268721

[R96] Romberg AR 2021. Vaping in the workplace: prevalence and attitudes among employed US adults. J Occup Environ Med. 63(1):10–17. 10.1097/JOM.0000000000002061 33105399 PMC7773161

[R97] Sahu R 2023. E-cigarettes and associated health risks: an update on cancer potential. Adv Respir Med. 91(6): 516–531. 10.3390/arm91060038 37987300 PMC10660480

[R98] Salazar MR 2025. Elevated toxic element emissions from popular disposable e-cigarettes: sources, life cycle, and health risks. ACS Cent Sci. 11(8):1345–1354. 10.1021/acscentsci.5c00641 40893954 PMC12395296

[R99] Sapru S 2020. E-cigarettes use in the United States: reasons for use, perceptions, and effects on health. BMC Public Health. 20(1):1518. 10.1186/s12889-020-09572-x 33032554 PMC7545933

[R100] Schmidt S 2020. Vaper, beware: the unique toxicological profile of electronic cigarettes. Environ Health Perspect. 128(5):52001. 10.1289/EHP6628 32363917 PMC7263459

[R101] Shehata SA 2023. Vaping, environmental toxicants exposure, and lung cancer risk. Cancers. 15(18):4525.), 10.3390/cancers15184525 37760496 PMC10526315

[R102] Shittu AAT 2022. Changes in e-cigarette and cigarette use during pregnancy and their association with small-for-gestational-age birth. Am J Obstet Gynecol. 226(5):730.e1–730.e10. 10.1016/j.ajog.2021.11.1354 PMC10329371 34864040

[R103] Sleiman M 2016. Emissions from electronic cigarettes: key parameters affecting the release of harmful chemicals. Environ Sci Technol. 50(17):9644–9651. 10.1021/acs.est.6b01741 27461870

[R104] Son Y 2020. Indoor air quality and passive e-cigarette aerosol exposures in vape-shops. Nicotine Tob Res. 22(10):1772–1779. 10.1093/ntr/ntaa094 32445475 PMC7542645

[R105] Soneji S 2017. Association between initial use of e-cigarettes and subsequent cigarette smoking among adolescents and young adults: a systematic review and meta-analysis. JAMA Pediatr. 171(8):788–797. 10.1001/jamapediatrics.2017.1488 28654986 PMC5656237

[R106] Soule EK 2023. Secondhand electronic cigarette aerosol in vehicles impacts indoor air quality. Drug Alcohol Depend. 250:110889. 10.1016/j.drugalcdep.2023.110889 37478503 PMC10528711

[R107] Sponziello M 2014. Epigenetic-related gene expression profile in medullary thyroid cancer revealed the overexpression of the histone methyltransferases EZH2 and SMYD3 in aggressive tumours. Mol Cell Endocrinol. 392(1–2):8–13. 10.1016/j.mce.2014.04.016 24813658

[R108] Sun Y 2024. Vaping: public health, social media, and toxicity. Online J Public Health Inform. 16:e53245. 10.2196/53245 38602734 PMC11046396

[R109] Sweet L 2019. Quitting behaviors among dual cigarette and e-cigarette users and cigarette smokers enrolled in the tobacco user adult cohort. Nicotine Tob Res. 21(3):278–284. 10.1093/ntr/nty222 30346585 PMC6379027

[R110] Szukalska M 2021. Toxic metals in human milk in relation to tobacco smoke exposure. Environ Res. 197: 111090. 10.1016/j.envres.2021.111090 33798522

[R111] Tang X 2021. Emissions from heated terpenoids present in vaporizable cannabis concentrates. Environ Sci Technol. 55(9):6160–6170. 10.1021/acs.est.1c00351 33825441

[R112] Temourian AA 2022. Why do smokers use e-cigarettes? A study on reasons among dual users. Prev Med Rep. 29:101924. 10.1016/j.pmedr.2022.101924 35911573 PMC9334340

[R113] Tobacco Monitoring. n.d. Monitoring U.S. E-cigarette sales: national trends. https://tobaccomonitoring.org/national/

[R114] Toledo EFV 2025. A comprehensive review of the harmful compounds in electronic cigarettes. Toxics. 13(4):268. 10.3390/toxics13040268 40278584 PMC12031152

[R115] Truth Initiative. 2022. A toxic, plastic problem: e-cigarette waste and the environment. https://truthinitiative.org/research-resources/harmful-effects-tobacco/toxic-plastic-problem-e-cigarette-waste-and-environment

[R116] U.S. Food and Drug Administration. 2024. Programmatic environmental assessment for electronic nicotine delivery system (ENDS) disposal regulations. https://www.fda.gov/media/179933/download

[R117] U.S. Food and Drug Administration. n.d. E-cigarettes, vapes and other electronic nicotine delivery systems (ENDS). https://www.fda.gov/tobacco-products/products-ingre-dients-components/e-cigarettes-vapes-and-other-electronic-nicotine-delivery-systems-ends

[R118] United States Environmental Protection Agency. 2025a. Summary of the Resource Conservation and Recovery Act (RCRA). https://www.epa.gov/laws-regulations/summary-resource-conservation-and-recovery-act

[R119] United States Environmental Protection Agency. 2025b. E-cigarettes, vapes, and other electronic nicotine delivery systems (ENDS). https://www.fda.gov/tobacco-products/products-ingredients-components/e-cigarettes-vapes-and-other-electronic-nicotine-delivery-systems-ends

[R120] Ussher M, Fleming J, Brose L. 2024. Vaping during pregnancy: a systematic review of health outcomes. BMC Pregnancy Childbirth. 24(1):435. 10.1186/s12884-024-06633-6 38902658 PMC11191278

[R121] Vaglenova J 2008. Long-lasting teratogenic effects of nicotine on cognition: gender specificity and role of AMPA receptor function. Neurobiol Learn Mem. 90(3): 527–536. 10.1016/j.nlm.2008.06.009 18662793

[R122] Vas CA, Porter A, McAdam K. 2019. Acetoin is a precursor to diacetyl in e-cigarette liquids. Food Chem Toxicol. 133:110727. 10.1016/j.fct.2019.110727 31377138

[R123] Vilcassim MJR 2023. Electronic cigarette use during pregnancy: is it harmful? Toxics. 11(3):278. 10.3390/toxics11030278 36977043 PMC10058591

[R124] Wang TW 2020. E-cigarette use among middle and high school students - United States, 2020. MMWR Morb Mortal Wkly Rep. 69(37):1310–1312. 10.15585/mmwr.mm6937e1 32941408 PMC7498174

[R125] Wang X, Lee NL, Burstyn I. 2020. Smoking and use of electronic cigarettes (vaping) in relation to preterm birth and small-for-gestational-age in a 2016 U.S. national sample. Prev Med. 134:106041. 10.1016/j.ypmed.2020.106041 32105682

[R126] Wang Y 2023. The association of current exclusive e-cigarette use and dual use of e-cigarettes and cigarettes with psychological distress among U.S. adults. Prev Med Rep. 36:102425. 10.1016/j.pmedr.2023.102425 37810268 PMC10556823

[R127] Woo W 2024. Ozonolysis of terpene flavor additives in vaping emissions: elevated production of reactive oxygen species and oxidative stress. Chem Res Toxicol. 37(6):981–990. 10.1021/acs.chemrestox.4c00051 38776470 PMC11187633

[R128] Zavala-Arciniega L 2021. Profile and patterns of dual use of e-cigarettes and combustible cigarettes among Mexican adults. Salud Publica Mex. 63(5):641–652. 10.21149/12365 35099887 PMC8827615

[R129] Zhang Y, Sumner W, Chen DR. 2013. In vitro particle size distributions in electronic and conventional cigarette aerosols suggest comparable deposition patterns. Nicotine Tob Res. 15(2):501–508. 10.1093/ntr/nts165 23042984

